# Early Interceptive Correction for Anterior Crossbite Using a Removable Appliance: A Pediatric Case Study

**DOI:** 10.7759/cureus.56072

**Published:** 2024-03-13

**Authors:** Asim Almarhoumi, Malak M Alwafi

**Affiliations:** 1 Department of Preventive Dental Sciences, Taibah University, Madinah, SAU

**Keywords:** mixed dentition, pediatirc dentistry, removable orthodontic appliance, interceptive orthodontics, crossbite

## Abstract

Anterior crossbite is a malocclusion that occurs for a variety of reasons, causes significant issues, and may be corrected in a variety of ways. Early recognition and timely intervention are crucial in managing anterior dental crossbites during the mixed dentition stage. The purpose of this report is to assist general dentists and pediatric dentists in distinguishing between cases within their scope of practice and those requiring referral to orthodontists and treating this condition immediately to prevent future complex treatment and improve patient aesthetics and function. This case report highlights the treatment of an eight-year-old patient with class III incisors on a skeletal class I base, presenting with an anterior crossbite. The patient was treated successfully using an upper removable appliance, showcasing a non-invasive and effective approach to correcting dental malocclusions early. The rapid correction of the crossbite within six weeks underscores the importance of early intervention and the potential for general dentists and pediatric dentists to manage such conditions efficiently, improving patient outcomes in aesthetics and function.

## Introduction

Dental practitioners must possess extensive knowledge and skills to diagnose and manage malocclusion in early mixed-dentition patients with developing dentition. This is crucial for implementing simple yet effective measures to eliminate or reduce the severity of malocclusion.

Interceptive orthodontics is a procedure used to simplify orthodontic care or correct a developing malocclusion. Richardson defined it in 1982 as treating problems early on, which can lead to better stability, function, and appearance, resulting in less time for treatment and better results with simple mechanics later on [1].

Early orthodontic treatment has been shown to have a positive impact on self-confidence, body image, and lifestyles of young children, as well as reducing the severity of developing malocclusion [2]. Research indicates that simple interceptive treatment can fully correct developing problems in mixed dentition in 15% of cases and improve the situation in 49% of cases [3]. This highlights the importance of early intervention in orthodontic issues to address concerns at a stage where corrections can be more effective.

According to the American Association of Orthodontists, a crossbite is an abnormal relationship of one or more teeth to the corresponding opposing teeth, causing buccolingual and labiolingual discrepancies in tooth relationships. Anterior crossbites are common malocclusions during the primary dentition period and at the beginning of mixed dentition, with an estimated prevalence of approximately 4-5% [4,5].

Etiologically, anterior crossbites can be attributed to skeletal, dental, or a combination of these factors. Anterior skeletal crossbites are often associated with skeletal discrepancies such as mandibular prognathism or mid-face deficiency. In contrast, anterior dental crossbites are dental malocclusions characterized by an abnormal axial inclination of the maxillary anterior teeth. They typically result from the palatal malposition of the maxillary incisors, leading to a lingual eruption path. Other factors that can cause this include injuries to the primary maxillary incisors that move permanent tooth buds to the back of the mouth, having extra front teeth, incisor crowding, biting your upper lip, deciduous teeth or roots that are too stuck in or dead, primary incisors that do not fall out properly, and odontomas [6,7].

Differentiating between dental and skeletal anterior crossbites is essential to determining the most suitable treatment. This can be accomplished by aligning the jaw in a centric relationship and assessing the relationship between the incisors and molars. Dental correction can be pursued if the incisors are in an edge-to-edge alignment and the molars are in a Class I relationship [8,9].

Correction of anterior dental crossbites is a common orthodontic treatment that can be achieved using removable or fixed appliances. This therapy has been shown to enhance dentofacial aesthetics and functional occlusion, in addition to preventing abnormal abrasions, anterior tooth fractures, and periodontal issues [10]. Furthermore, the presence of an anterior crossbite with mandibular displacement may exacerbate temporomandibular disorder (TMD) in individuals predisposed to the condition [11,12]. This is supported by two recent randomized controlled clinical trials on anterior crossbite treatment, where they found an increase in the condylar volume after interception, indicating a positive impact on the temporomandibular joint [13,14].

Diagnosing and intercepting malocclusions before they worsen is crucial to preventing complications [15]. The mixed dentition phase provides a valuable opportunity to assess and identify developing malocclusions and provide necessary treatment.

## Case presentation

**Figure 1:**
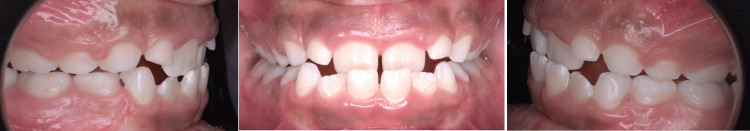
Pretreatment intraoral photographs showing the anterior crossbite affecting the maxillary central incisors.

An eight-year-old Saudi girl presented with Class III incisors on a skeletal Class I base, with average vertical proportions. Her primary concern was the displeasing positioning of her maxillary central incisors, which were situated behind her lower anterior teeth. The patient had no significant medical or dental history, no allergies, was not taking any medications, and had no family history of Class III malocclusion. Furthermore, the maxillary lateral incisors had not yet erupted, and both the permanent maxillary right and left central incisors were in crossbite (Figure 1).

The patient exhibited a Class I molar relationship on both sides, a 1 mm reversed overjet, and a 2 mm overbite. She was in the early mixed-dentition phase.

In the maxillary arch, there was no spacing, and sufficient mesiodistal space was available for the maxillary central incisors to move forward labially. The maxillary and mandibular dental midlines coincided with the facial midline. The treatment objective for the patient included correcting the anterior crossbite, restoring a normal overbite and overjet, realigning the patient's anterior teeth for an acceptable inclination, and enhancing facial and dental aesthetics.

The pediatric patient received guidance on maintaining proper dental hygiene, which included supervised brushing twice daily (after breakfast and before bedtime, considered the most crucial brushing times). The recommended brushing duration was set at two minutes. Additionally, to ensure the cleanliness of their removable appliances, they were advised to brush them with soap.

**Figure 2:**
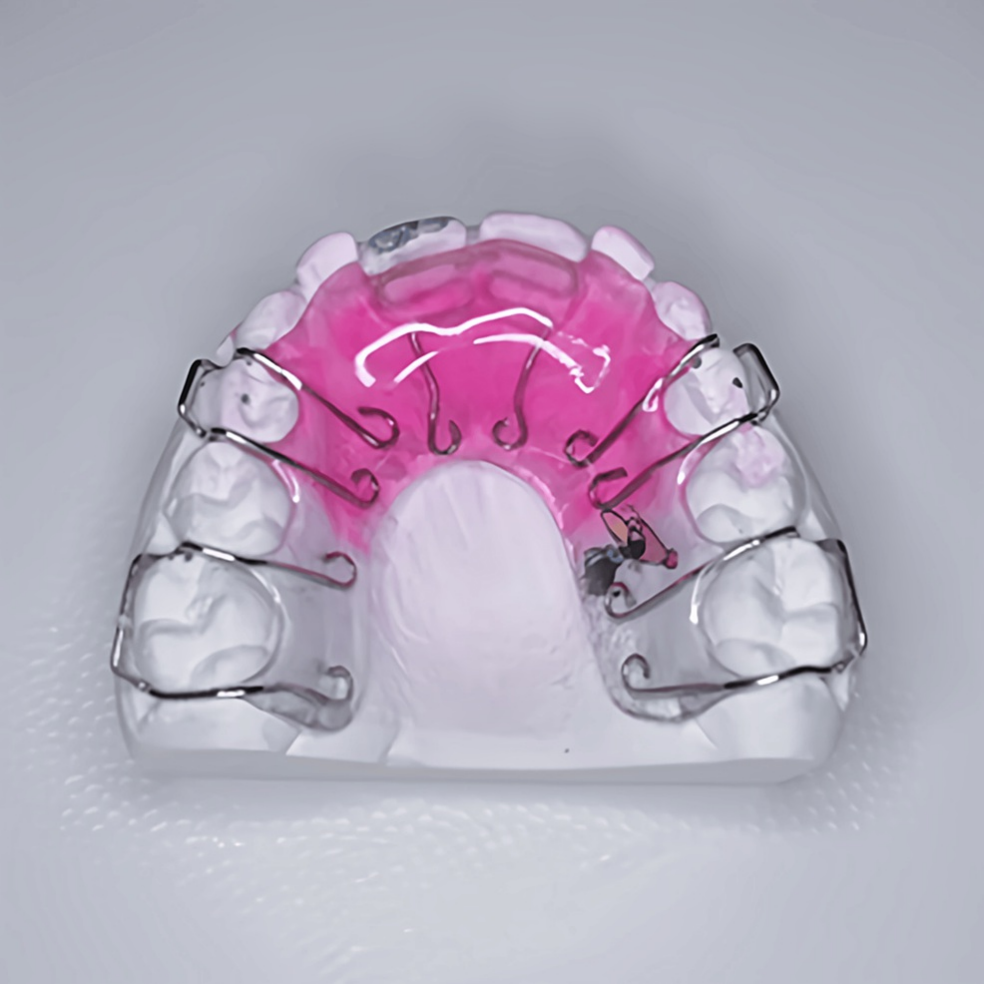
Upper removable acrylic appliance design; Z-springs palatal to the upper central incisors (active components), and Adams clasps on the primary and permanent molars (Retention).

The selected appliance for correcting the anterior crossbite was an upper removable appliance designed as follows: two Z-springs made of 0.5 mm stainless steel round wire (active components), Adam’s clasps engaging the mesial and distal cervical undercuts of the upper first primary and permanent molars bilaterally, made of 0.6 mm and 0.7 mm stainless steel round wires, respectively (retentive components). An acrylic base plate was incorporated to integrate these components into a single functional unit. This base plate also contributed to anchoring and retaining the device in the oral cavity (Figure 2).

The appliance was activated by slowly pulling the active parts labially. This adjustment, measuring 2 mm, caused the teeth to move 1 mm labially.

Afterward, it was carefully positioned in the mouth, ensuring that the Z-spring arms were in contact and parallel against the palatal surfaces of the central incisors affected by the crossbite, thus facilitating labial tooth movement. Following this, the patient received instructions on how to insert and remove the appliance and was advised to wear it continuously, including during meals and sleep, until correction was fully achieved.

**Figure 3:**
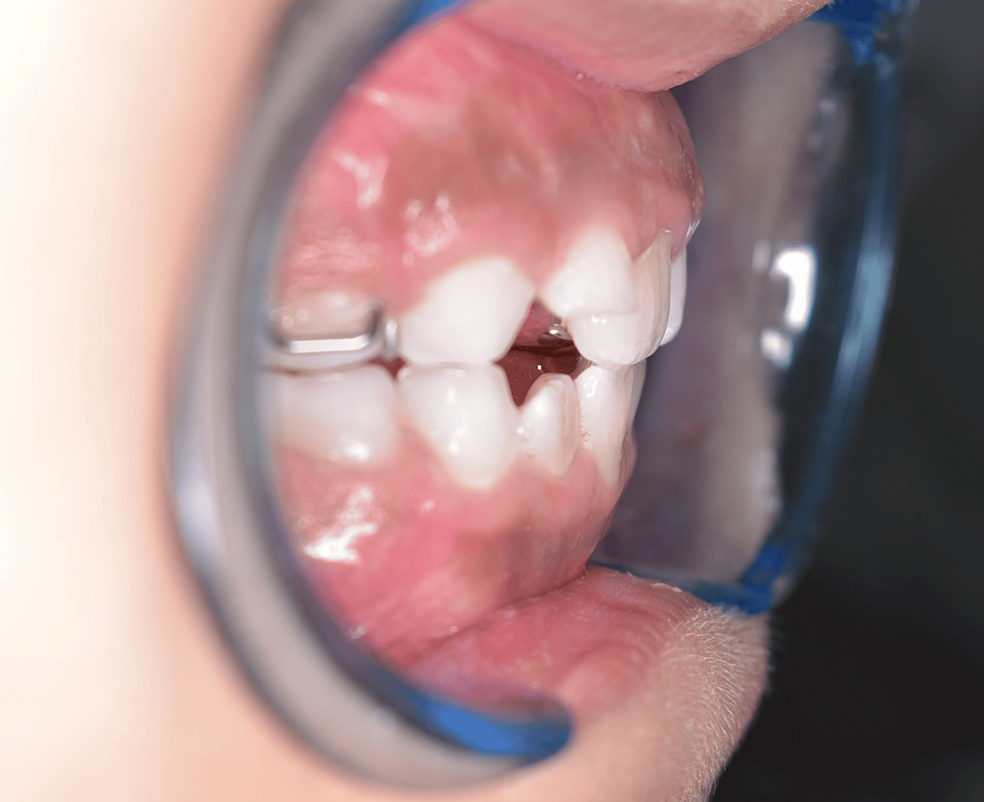
Incisors showing an edge-to-edge relationship.

Over a span of four weeks, the Z-springs were activated every seven days. The establishment of an edge-to-edge bite relationship between the maxillary and mandibular incisors (Figure 3) remarkably shows that the correction of the crossbite was successful within an additional two weeks.

**Figure 4:**
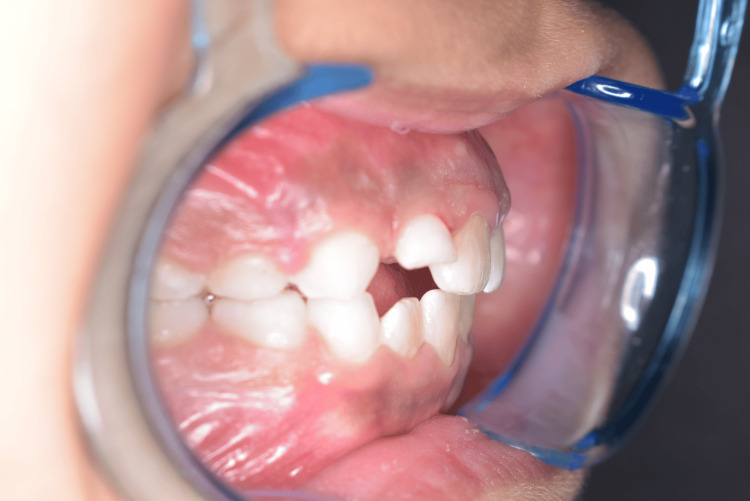
The incisors showing a positive overjet after six weeks.

All maxillary incisor crossbites were successfully corrected after the subsequent six weeks of active treatment, and no issues were observed during the six weeks of clinical and radiographic follow-up (Figure 4).

**Figure 5:**
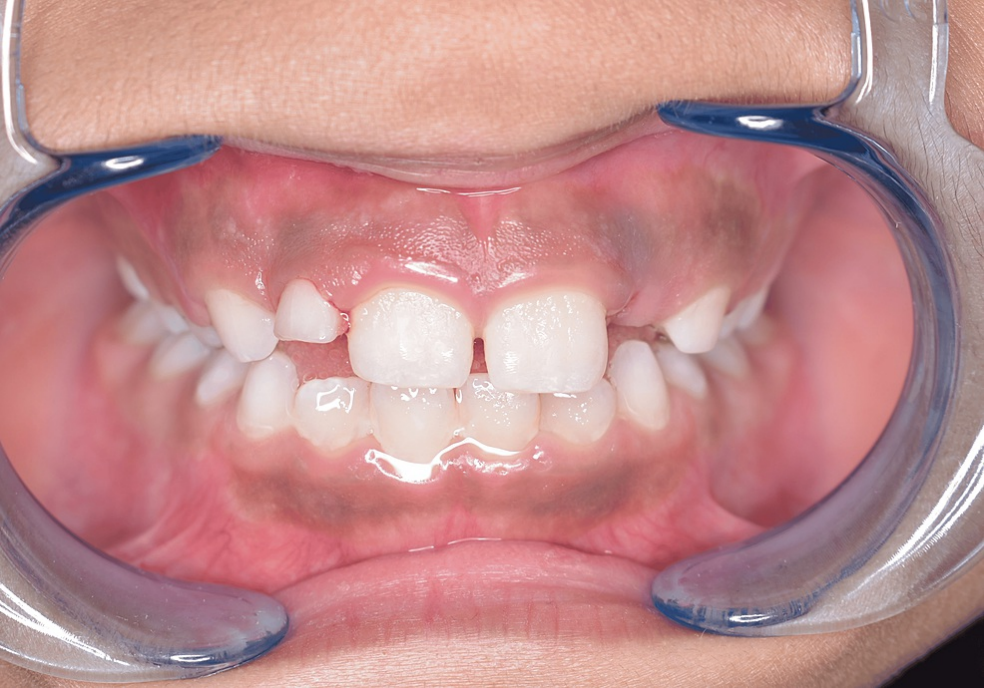
End of anterior crossbite treatment, positive overjet, and overbite are established.

After achieving a Class I incisor relationship, an overcorrection by an extra 1 mm of anterior tipping was executed to minimize the risk of relapse. Furthermore, maintaining a positive and stable overbite between the upper and lower incisors was deemed crucial for preserving the achieved correction (Figure 5).

## Discussion

Maintaining or enhancing arch integrity to facilitate the emergence of permanent teeth and prevent the development of more complex malocclusion is one of the fundamental objectives of orthodontic treatment. Anterior dental crossbite is not an uncommon malocclusion that can cause aesthetic and functional concerns for both children and parents, and it rarely resolves naturally [16].

If multiple teeth are in crossbite or there is a reverse overjet without functional displacement, it could indicate a true Class III skeletal problem, or a true malocclusion. A specialist referral is needed to assess the extent of the discrepancy [16]. For significant antero-posterior skeletal discrepancies, functional appliances, chin cups, or maxillary protraction devices may be employed to modify growth. Maxillary protraction with facemask therapy is most effective in early mixed dentition, so early referral is recommended [17]. The optimal time to correct an anterior crossbite is between ages 8 and 11, when the tooth is actively erupting and the root is forming [14,17].

A pseudo-Class III occlusion is an anterior crossbite without a skeletal discrepancy, caused by factors like dental crowding and premature occlusal contact. This condition can lead to functional forward movement of the mandible during closure, resulting in an anterior crossbite. The maxillary incisors typically exhibit retroclination, while the mandibular incisors are proclined. Identifying a pseudo-Class III occlusion involves achieving an edge-to-edge position or normal overjet. Neglecting to correct the crossbite early may make it more challenging and require a more comprehensive approach, potentially leading to a skeletal Class III malocclusion [15].

The literature offers descriptions of various types of appliances used for early treatment of pseudo-Class III malocclusion. These options include fixed and removable orthodontic appliances, tongue blades, composite inclined planes, reversed stainless steel crowns [18]. When selecting a treatment approach, it's crucial to consider factors such as the patient's age, stage of dental development, the number of affected teeth, the severity of the overbite, and the motivation of both the child and the parents for the procedure [10].

In this particular case, a removable orthodontic appliance was the treatment of choice for several reasons. First, the appliance was chosen based on patient-specific factors such as age, occlusal analysis, and skeletal development, making it suitable for use in the early mixed-dentition phase. Second, it provides another safe, simple, and aesthetically pleasing option for treating an anterior crossbite. In this case, three primary advantages were observed: (1) These appliances are fabricated in a laboratory, reducing chair time; (2) they can be easily removed for special social occasions when having wires on the facial aspect of the teeth is undesirable; (3) Their design allows for easy cleaning, promoting good oral hygiene. The spring within the appliance can be adjusted to control the necessary tooth movement, making it easier to manage and reducing the risk of displacement.

Removable appliances are a cost-effective and easy-to-manage treatment option for anterior crossbite, especially in pediatric patients or those in the mixed dentition stage. They are more cost-effective than fixed appliances and can be beneficial in areas with limited access to dental care or financial constraints. Patient compliance can be managed when patients are motivated and understand the importance of the treatment. The design and fabrication process of removable appliances with active components is straightforward, involving steps such as drawing the design, positioning clasps and retentive components, and acrylic base plates [19].

Throughout the treatment process, the patient reported minimal discomfort. The treatment successfully corrected the malocclusion and resulted in an aesthetically pleasing smile. Based on these outcomes, a removable device with a labiolingual spring may be the primary treatment choice for correcting a crossbite involving multiple incisor teeth.

Another and the most basic method for treating an anterior crossbite is the tongue blade, which the patient is advised to use during periods of relaxation. In this method, the incisal edges of the mandibular teeth act as a fulcrum to absorb the reciprocal lingual pressures, and the biting force is applied to the lingual face of the corresponding maxillary tooth to encourage its forward movement. However, this approach is often insufficient when more than one tooth is involved, and it is more cumbersome to use by patients as it doesn’t provide sufficient duration for force application compared to other methods [7].

An alternative, effective, and non-invasive treatment option is a composite inclined bite plane, which is the preferred choice in some cases. It is important to note, though, that a composite plane may not be used if the anterior crossbite extends beyond one-third of the crown length. Furthermore, the cement used with this type of appliance has been associated with the development of gingivitis [18].

Another treatment option involves using a prefabricated stainless steel crown that has been reversed to address the crossbite. The primary drawback of this method is the challenge of adapting a prefabricated crown to fit the tooth in crossbite accurately [20]. Additionally, many children and their families reject this treatment option due to its less aesthetically appealing appearance [21].

## Conclusions

The successful treatment of anterior crossbite malocclusion in the early stages, as demonstrated in this case, emphasizes the significance of early intervention to avoid complex future treatments. It showcases that with the appropriate appliance, chosen based on patient-specific factors such as age, soft tissue analysis, and maxillary development, general dentists and pediatric dentists can effectively manage mild dental crossbites. This approach not only prevents the progression of malocclusion but also supports the natural growth patterns of the maxilla and mandible, aligning them in their normal relationship. Ultimately, this case serves as a practical guide for dental professionals in determining when simple appliances can be utilized within general practice and when referral to an orthodontist is necessary for more severe cases.
